# Molecular architecture of monkeypox mature virus

**DOI:** 10.1038/s41421-024-00741-5

**Published:** 2024-10-22

**Authors:** Ye Hong, Baoying Huang, Junxia Zhang, Cheng Peng, Weizheng Kong, Wenjie Tan, Sai Li

**Affiliations:** 1 Beijing Frontier Research Center for Biological Structure & Tsinghua-Peking Center for Life Sciences & State Key Laboratory of Membrane Biology, School of Life Sciences, Tsinghua University, Beijing, China; 2 National Key Laboratory of Intelligent Tracking and Forecasting for Infectious Diseases, NHC Key Laboratory of Biosafety, National Institute for Viral Disease Control and Prevention, Chinese Center for Disease Control and Prevention, Beijing, China

**Keywords:** Cryoelectron tomography, Cryoelectron microscopy

Dear Editor,

The mpox outbreak since 2022 has caused over 106,310 lab-confirmed infected cases with 234 deaths across 123 countries/territories/areas and was declared a public health emergency of international concern (PHEIC) by the World Health Organization in 2022 and again in 2024. The causative pathogen, monkeypox virus (MPXV), is a zoonotic double-stranded DNA (dsDNA) orthopoxvirus encoding 181 proteins^1^. Although smallpox vaccines based on vaccinia virus (VACV) could provide cross-protection against MPXV infection and have been used for current mpox prevention, no licensed specific vaccine or medicine against MPXV is available globally^2^.

Despite efforts to elucidate the MPXV genome uncoating and replication mechanisms using recombinant proteins, in situ structural insights into major structural proteins and assembly of the authentic MPXV particles are missing. Early electron microscopy (EM) micrographs of the negative-stained or resin-embedded MPXV mature virus (MV), the basic form of infectious MPXV particles, have revealed its overall architecture comprising a lipid envelope coated with proteins, two lateral bodies and a biconcave viral core encapsulating genomic DNA^3^. Following fusion between MV envelope and cell membrane, lateral bodies will detach from the viral core and deliver effector proteins to host cytosol, while the viral core will be released and function as an early transcription factory protecting the virus genome^4^. Recent progress in cryo-electron tomography (cryo-ET) has enabled the structural study of authentic virus with molecular level details^5^. Studies applying cryo-ET on VACV have provided in-situ structural details of orthopoxviral core, revealing that protein A10 trimers form a layer of palisade, A4 likely decorates the exterior of A10 trimers and A3 likely forms the inner wall^6–8^. Besides, multiple copies of flower-shaped portal complex composed of a portal and six surrounding A10 trimers were observed^6–8^. However, multiple amino acid differences exist between the major structural proteins of VACV and MPXV and the currently circulating MPXV carries multiple mutations in assembly-related major structural proteins such as L4 and A10, suggesting possible variation in virus morphology^9^. Here, we optimized an MPXV propagation and purification workflow. Combining cryo-ET and sub-tomogram averaging (STA), we determined the structures and assembly of palisade proteins and portal complexes to unveil the architecture of intact MPXV MVs. We also discovered irregularly-shaped MV particles with distorted core walls, which have not been reported before.

The first imported mpox case in mainland China was confirmed on September 16, 2022^10^, and the first locally acquired mpox case in mainland China was reported in Beijing on May 31, 2023^11^. The MPXV strain MPXV-B.1-China-C-Tan-CQ01, which phylogenetically belongs to the B.1 lineage branch of IIb clade (or West African clade), was isolated from skin blister fluid of the first imported mpox case in mainland China (National Pathogen Resource Center of China preservation ID: China-CQ202209) and used for structural analysis. Briefly, virions were propagated in Vero cells in biosafety level 3 (BSL-3) laboratories, chemically fixed by paraformaldehyde (PFA), and transferred to a BSL-2 laboratory for isolation and plunge-freezing. Since the majority of MPXV MVs remain intracellular, it is necessary to lyse the infected cells before isolation to obtain sufficient virions for imaging. However, cell lysis by sonication is prohibited in most BSL-3 labs, and chemical fixation prior to cell lysing would cross-link the virions with cellular contents. As an alternative, we adjusted conventional purification method to yield MVs of high purity and concentration suitable for cryo-ET imaging (Supplementary Fig. S1). The tomographic reconstructions indicated that MV particles prepared using this method remained intact and were morphologically consistent with naturally released extracellular MVs (Supplementary Fig. S2).

A total of 210 tomograms of MPXV MVs were imaged and reconstructed, revealing an oblate ellipsoidal shape of these virions (Supplementary Table S1). Among them, ~60% comprise a membrane protein-coated lipid envelope packaging a closed biconcave viral core and two lateral bodies, resembling the previously imaged MPXV^3^ and VACV^6,12^ MVs (Fig. 1a–d; Supplementary Video S1). In comparison to the more rectangularly-shaped VACV MV^6^, these MPXV MVs have significantly shorter long axis and longer intermediate axis (*P* < 0.0005), exhibiting an ellipsoidal shape with uniform dimensions of ~313 × 267 × 236 nm (Fig. 1e), while VACV MVs have dimensions of ~347 × 260 × 240 nm^6^. STA of palisades on viral cores revealed an 11.45-Å resolution trimer with dimensions of ~8 × 13 nm (width × height) (Fig. 1f; Supplementary Figs. S3, S4). The map fits globally well with an AF2Complex-predicted trimer of the MPXV A10 residues 1–614 (Fig. 1f; Supplementary Fig. S5a), but exhibited local differences as described below. On the top transverse plane, each two flanks (A1-B3, A2-B1, A3-B2) were connected by unassigned densities at flanks A labeled by green arrowheads (Supplementary Fig. S5b). We speculate that these extra densities belong to other proteins interacting with A10, such as A4^6,13^. On the central part, residues 90–107 of each MPXV A10 subunit are predicted to form an intramolecular connection (Supplementary Fig. S5c) similar to the previously reported VACV palisade structures^6–8^ (Supplementary Fig. S5d). However, no such connecting or extending density is visible in the MPXV palisade map. We speculate that the structural variation is related to the D98N mutation in A10 carried by the circulating clade IIb MPXV^9^. This mutation is located right in the interacting surface between two subunits and would change the local charges from negative to neutral (Supplementary Fig. S5c, d). Besides the variation in central slice, MPXV palisade is more upright and constricted, while VACV palisade is more twisted and open (Supplementary Fig. S5e).

**Fig. 1:**
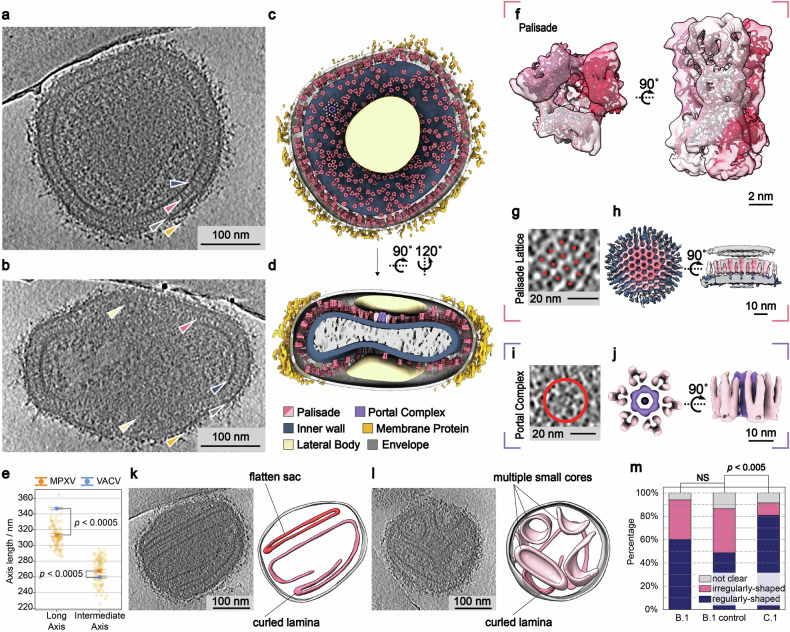
Molecular Architecture of MPXV MV. **a**–**d** Tomogram slices showing the top view (**a**) and side view (**b**) of two intact MV particles. Arrowheads indicate the inner wall (dark blue), palisade (pink), envelope (gray), membrane protein (orange) and lateral bodies (light yellow). Top view (**c**) and side view (**d**) of a composite structure of the MV displayed in **a** reconstructed by projecting structures of portal complexes and palisade trimers together with envelope, membrane protein, inner wall, virion interior, and lateral bodies segmented from their corresponding densities. Palisade and portal complex particles with low cross correlation value or obvious misalignment were removed. Colors corresponding to various viral components are labeled in **d**. **e** Dimensional statistics of typical MPXV MV particles (*n* = 85). Measurement of each MPXV MV is indicated by transparent orange dot. Opaque dots indicate average axis length and error bars indicate standard error of mean. Statistics of VACV from reference^6^ is plotted in blue. **f** The palisade trimer structure map fitted with an AF2Complex-predicted trimer of A10 residues 1–614. Different colors indicate three subunit of palisade trimer. **g** A tomogram slice with 5.44 nm thickness showing the honeycomb-like palisade lattice on viral core. Individual palisades are marked with red dots to show their relative arrangement. **h** Structure of the honeycomb-like lattice assembled by the palisades on viral core. The central lattice is labeled with deep pink, while the lateral palisades out of alignment mask are labeled in lighter pink. **i** A tomogram slice with 16.32 nm thickness showing the flower-shaped portal complex (marked with a red circle) identified on viral core. The tomogram was tilted to show top view of the portal complex. **j** Structure of the portal complex. The palisades are colored in light pink and the portal in purple. **k**, **l** Tomogram slices showing two exemplary irregularly-shaped MPXV MV particles. The envelope (gray) and core walls (pink) are segmented from the corresponding densities in the tomograms to show the irregular assembly of viral core walls. **m** Percentage of regularly-shaped and irregularly-shaped virions in MPXV-B.1-China-C-Tan-CQ01 prepared via method A (B.1, *n* = 186), MPXV-B.1-China-C-Tan-CQ01 prepared via method B (B.1 control, *n* = 37) and MPXV-C.1-China-C-Tan-BJ01 prepared via method B (C.1, *n* = 47). NS, not significant. All tomograms shown here were lowpassed to 80 Å. Thickness of tomogram slices is 27.2 nm unless stated differently.

Next, we investigated the assembly of viral cores. Similar to VACV palisade^7,8^, the bottom of MPXV palisade is predominantly covered with positive charges (Supplementary Fig. S6a). This could facilitate palisades’ electrostatic interaction with the underlying inner wall proteins, which is reflected by the relatively higher local resolution at the palisade bottom (Supplementary Fig. S6b). Classification with a larger mask indicated that 63% palisade trimers assemble into honeycomb-like lattice with an average center-to-center distance of 9 nm (Fig. 1g, h; Supplementary Fig. S3), while the remaining palisades were less ordered (Supplementary Fig. S3). Apart from the palisade, flower-shaped portal complexes were occasionally observed on the top and bottom surface of the MPXV viral core with no obvious distribution pattern (Fig. 1c, d, i). STA of these portal complexes revealed a density map consisting of six palisade trimers surrounding a hexagonal-cylindrical portal (Fig. 1j; Supplementary Fig. S3). The average center-to-center distance of palisade proteins surrounding the portal is 10 nm (Supplementary Fig. S7a), larger than that in the honeycomb-like lattice and possibly causing local distortion of palisade distribution. The portal lumen has an inner diameter of 6 nm on the narrowest top part and 9 nm on the widest central part (Supplementary Fig. S7b). A rod-shaped density could be observed in the lumen (Fig. 1i, j; Supplementary Fig. S7). To illustrate the MPXV architecture, we reconstructed a composite structure of an exemplary MPXV MV (Fig. 1c, d; Supplementary Video S1). Notably, only particles with relatively high confidence were projected to the reconstruction, while the misaligned particles were removed.

Interestingly, some virions are irregularly-shaped and exhibit significantly different interior features compared to the previously reported MPXV MVs^3^ or wild-type VACV MVs^6–8^. The core walls of these particles present in forms of flatten sacs, curled laminae, or multiple small cores (Fig. 1k, l, Supplementary Videos S2, S3). Among all virions imaged unbiasedly (*n* = 186), 33.9% are irregularly-shaped (B.1 in Fig. 1m). To verify if the irregularly-shaped particles originated from sample preparation, we analyzed the tomograms of extracellular MVs, which were released naturally and had not been treated with detergent or sonication. In these control particles, similar percentage of irregularly-shaped virions was also observed (Fig. 1m, Supplementary Fig. S8a), suggesting a native feature of the core wall. MPXV clade IIb circulating since 2022 further evolved into multiple branches. To verify whether this phenotype is specific to MPXV-B.1-China-C-Tan-CQ01, we isolated and propagated another MPXV strain from the C.1 lineage, MPXV-C.1-China-C-Tan-BJ01, which was isolated from a local case in Beijing in 2023 (hMpxV/China/BJCDC-01/2023). The strain was propagated and purified using the same method as for MPXV-B.1-China-C-Tan-CQ01. Cryo-ET revealed that it also contains irregularly-shaped particles (10.6%, C.1 in Fig. 1m; Supplementary Fig. S8b). We speculate that the atypical core phenotype could be a characteristic of zoonotic infection and MPXV may not have perfectly adapted to a human host, as similar atypical internal structure was observed on modified vaccinia virus Ankara^14^.

In summary, here we report the molecular architecture of intact MPXV MVs. Apart from the typical MV particles, a class of irregularly-shaped MV particles packaging distorted core walls were observed in two representative MPXV strains isolated from the 2022 and 2023 infections. Further investigations are needed to understand their formation mechanism and correlation to virulence. Our work broadens the understanding of orthopoxvirus assembly and provides a new aspect for orthopoxvirus morphogenesis research.

## Supplementary information

Supplementary Information

Supplementary Video S1

Supplementary Video S2

Supplementary Video S3

## Data Availability

EM maps have been deposited in the Electron Microscopy Data Bank under accession codes EMD-61555, EMD-61556, and EMD-61557. Electron tomograms of representative virions have been deposited in the Electron Microscopy Data Bank under accession codes EMD-61558, EMD-61559, and EMD-61560.
