# Supplementary material for: Molecular architecture of monkeypox mature virus
Source: Cell Discov. 2024 Oct 22;10:108. doi: 10.1038/s41421-024-00741-5 (PMC11496496; doi:10.1038/s41421-024-00741-5)
Supplement: Supplementary file 1 — Supplementary Information [file 41421_2024_741_MOESM1_ESM.pdf]

Molecular Architecture of Monkeypox Mature Virus

Ye Hong[1,3], Baoying Huang[2,3], Junxia Zhang[1,3], Cheng Peng[1], Weizheng Kong[1], Wenjie Tan[2,*] and Sai Li[1,*]

[1]Beijing Frontier Research Center for Biological Structure & Tsinghua-Peking Center for Life Sciences & State Key Laboratory of Membrane Biology, School of Life Sciences, Tsinghua University, Beijing 100084, China

[2]National Key Laboratory of Intelligent Tracking and Forecasting for Infectious Diseases, NHC Key Laboratory of Biosafety, National Institute for Viral Disease Control and Prevention, Chinese Center for Disease Control and Prevention, Beijing 102206, China

[3]These authors contributed equally

[*]Correspondence: tanwj@ivdc.chinacdc.cn (W.T.), sai@tsinghua.edu.cn (S.L.)

Materials and Methods

MPXV Isolation and Propagation

MPXV isolation and propagation were performed in biosafety level 3 (BSL-3) laboratories of the Viral Disease Control and Prevention, China CDC. The MPXV strain MPXV-B.1-China-C-Tan-CQ01 was isolated from the first imported mpox case in mainland China[1]. The MPXV strain MPXV-C.1-China-C-Tan-BJ01 was isolated from the first local mpox case in mainland China[2]. Specimens were diluted at a 1:5 ratio in Minimum Essential Medium (MEM) supplemented with 2% fetal bovine serum and 1% antibiotics (penicillin 5,000 IU/mL, streptomycin 2,500 μg/mL, and amphotericin B 10 μg/mL, collectively referred to as PSA), and maintained at 25°C for 30 min before being inoculated into the Vero cells. After inoculation, the cells were incubated with the diluted specimens for 6 hours at 37°C in 5% CO_2, and subsequently cultured for 96 hours in refreshed medium. The isolated virus was identified through cytopathic effect observation, quantitative polymerase chain reaction and immunofluorescence assay. Plaque assays were utilized to quantify infectious plaque-forming units (PFUs).

For MPXV propagation, Vero cells were cultured in T-175 flasks and infected with a multiplicity of infection of 0.01, then cultivated in MEM containing 2% fetal bovine serum for four days at 37 °C in 5% CO_2. MPXV cultures were harvested once all cells exhibited cytopathic effect without significant detachment or flotation. Virus titers were determined by plaque assay, ranging between 2×10^6 and 1×10^7 PFU/ml.

MPXV Sample Preparation

To obtain sufficient MPXV MVs for cryo-ET imaging, infected cells were suspended in phosphate-buffered saline (PBS) and subjected to three freeze-thaw cycles (-80 ℃ to 37 ℃) to facilitate the release of intracellular virus particles. Subsequently, cell debris was removed by centrifugation at 4 ℃ and 2,000 × g for 10 minutes, and the released virus particles in the supernatant were fixed with 3% PFA at 4 ℃ for 48 hours within the BSL-3 laboratory.

Upon complete inactivation, the virions were transferred to a biosafety level 2 (BSL-2) laboratory for isolation. First, the virions were pelleted through a 36% sucrose cushion by ultracentrifugation (Beckman, IN) at 4 ℃ and 32,900 × g for 100 minutes. Next, the pellet was treated with 1% Triton X-100 at 37 ℃ for 2 h to alleviate inter-virion crosslinking, and concentrated by ultracentrifugation through 36% sucrose cushion. Subsequently, the sample was treated with a no-touch ultrasonic homogenizer (SCIENTZ, CN) at 80% power in core buffer (10 mM Tris-HCl pH 9.0, 1 mM DTT). The separated virus particles were then purified by 15%-45% sucrose-density gradient ultracentrifugation at 26,000 × g for 60 min and fractionated with a TRIAX flow cell (BioComp, CA). The band containing virions was collected, further treated with 1% NP-40 at 37 °C overnight, concentrated through ultracentrifugation at 32,900 × g for 60 min and finally resuspended in distilled water. The main procedure was shown in Fig. S1, method A.

Extracellular MVs that were naturally released were isolated directly from the infected cell supernatant. For MPXV-B.1-China-C-Tan-CQ01 strain, the sample was purified by ultracentrifugation first through 36% sucrose cushion, and then 15%-45% sucrose-density gradient. The virus fraction was diluted in PBS, sedimented at 32,900 × g for 60 min to remove sucrose and finally resuspended in distilled water (Fig. S1, method B). For MPXV-C.1-China-C-Tan-BJ01 strain, purification followed the same steps, except that density gradient centrifugation was not performed.

Cryo-electron Tomography

For cryo-ET sample preparation, purified virions were sonicated for 3 min and centrifuged at 5,000 rpm for 1 min to remove large aggregates. A glow-discharged 200 mesh holey carbon film coated copper grid (R2/2; Quantifoil, Jena, Germany) was soaked into 10 μL purified virus and incubated at room temperature for 60 min. Then, 4 μL distilled water was supplemented. The grid was blotted for 3 s and plunge frozen in liquid ethane using a Cryo-plunger 3 (Gatan, CA).

The grids were imaged using a 300 kV Titan Krios electron microscope (Thermo Fisher Scientific, Hillsboro, OR) equipped with GIF Quantum energy filter (slit width 20 eV) and K3 direct electron detector (Gatan, CA). Data for sub-tomogram averaging (STA) was collected under 64,000× magnification and super resolution mode, resulting in a calibrated pixel size of 1.36 Å. Tilt series were collected using the dose-symmetric scheme in SerialEM[3] with a tilt range from -51° to 51° and a step size of 3°. For each tilt, a movie consisting of 10 frames was recorded at an exposure of 3.94 $e^-/Å^2$, resulting in a total dose of 137.9 $e^-/Å^2$ per tilt series.

Cryo-electron Tomography Data Processing and Sub-tomogram Averaging

Tilt series of the detergent-treated MPXV-B.1-China-C-Tan-CQ01 MVs were collected and were preprocessed using an in-house developed software. In brief, beam-induced motion was corrected by averaging the last 8 frames of each movie using MotionCor[4] and MotionCor2[5]. Defoci of the averaged movies were estimated using GCTF[6]. Tilt series were then aligned using AreTomo 1.2.5[7], three-dimensional contrast transfer function (CTF) corrected and reconstructed using NovaCTF[8].

The viral core walls of typical MVs from 105 tomograms were manually segmented in Dynamo[9]. For STA, 21,266 particles were oversampled along the segmented palisade layer. Additionally, 3,339 top-view particles were manually selected in IMOD[10]. Initial alignment of the palisade lattice was performed in Dynamo[9] without symmetry imposed under 4-fold binning. After observing a clear C6-like palisade arrangement, C3 symmetry was applied for further alignment, resulting in a global STA density map illustrating a honeycomb-like palisade lattice structure.

To distinguish honeycomb-like lattice from less ordered palisade trimers, all particles underwent random in-plane rotation before being classified in Dynamo with two references created as described below. The honeycomb-like palisade lattice obtained above was C360 symmetrized and low-pass filtered to serve as a reference for the less ordered palisades. The central palisade trimer of the C360 symmetrized map was extracted as a single unit, and seven units were aligned to the honeycomb-like lattice to establish a reference for the honeycomb-like lattice structure.

For STA of single palisade trimer, the central palisade trimer of the above aligned lattice was masked and randomly rotated in-plane for further alignment with C3 symmetry imposed. After obtaining a solid density map, we cropped the single palisade trimer under 2-fold binning for finer alignment. The final map was reconstructed and sharpened with a b-factor of -1,766.47 Å^2 in Relion 4.0[11]. The map displayed in the figure was dust-hided and the mask used for sharpening was applied to it. After each oversampling project, geometric restriction was applied to remove outlying or misoriented particles. Resolution of palisade STA structure is estimated by Fourier Shell Correlation method with dynamo[9].

For STA of the portal complex, 42 top-view portal complexes were manually annotated in IMOD[10] for initial alignment in Dynamo[9], resulting in a preliminary map resembling a previously reported VACV portal complex structure (emd_18917)[12]. To obtain side-view particles from

oversampled particles, two independent multireference alignment projects were done to distinguish portal complex from palisade proteins. The first project classified portal complex particles from the palisade lattice under 4-fold binning, based on the reference of initially aligned top-view portal complex from manually picked particles. The second project classified portal complex particles from single palisade trimers under 2-fold binning, based on the reference of emd_18917. The particles classified as portal complexes in both classifications underwent further analysis. Those with a coordinate difference less than 10.88 nm and an orientation difference smaller than 30° between two alignments were extracted and combined with the manually picked particles for final alignment and average. The map displayed in the figure was segmented and its surrounding noise was masked to display with ChimeraX[13]. The summary of STA procedure is shown in Fig. S3.

Density Map Segmentation and Whole Virus Projection

Segmentation within density maps shown in figure 1h and 1j was done in ChimeraX[13]. Virus envelope, lateral bodies, inner wall and its interior densities were segmented manually from IsoNet[14]-processed tomograms using 3D Slicer[15]. Membrane proteins were identified automatically with ilastik[16] and density contacting virus envelope was selected for display with Amira[17]. Palisade trimers selected for 2-fold binning alignment and manually picked portal complexes were projected and displayed using an in-house Matlab script and connnverted into ChimeraX[13]. All segmented components and projected proteins were imported ito ChimeraX[13] to reconstruct a virion model.

AF2Complex Prediction

Atomic model used in fitting was predicted by AF2Complex 1.4[18]. Residues 1-614 of MPXV-B.1-China-C-Tan-CQ01 A10 was predicted as a trimer, and residues 1-281 of MPXV-B.1-China-C-Tan-CQ01 A4 was predicted as a monomer.

Quantification and Statistical Analysis

The long axis and intermediate axis of MPXV MVs were measured in IMOD[10]. MPXV dimensions were compared with VACV dimensions via Weltch's t-test. All VACV dimensions were analyzed based on the mean value, standard error of mean and event number of VACV measurements previously reported by M. Hernandez-Gonzalez. *et al.*[12] Comparisons between dimensions of MPXV MVs purified via two methods shown in Figure S2b were performed in Python.

Statistical test of regularly-shaped and irregular-shaped virus particles is done in python based on binomial distribution hypothesis test. Data used to compare two strains are collected unbiasedly.

Statistical plots were generated in Python unless stated otherwise.

Supplementary Figures

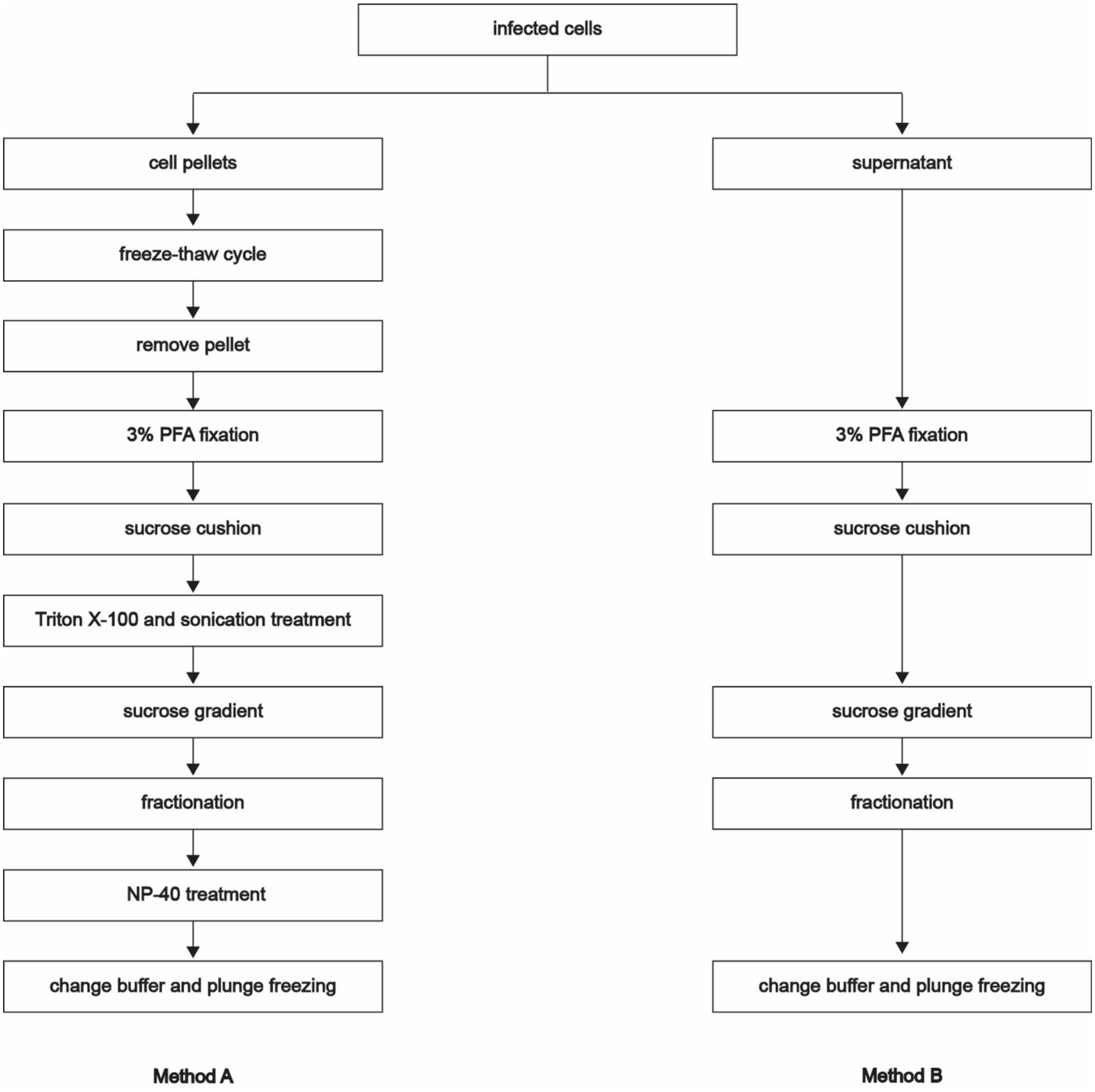

Fig. S1 Purification workflow of MPXV-B.1-China-C-Tan-CQ01 strain MV particles.

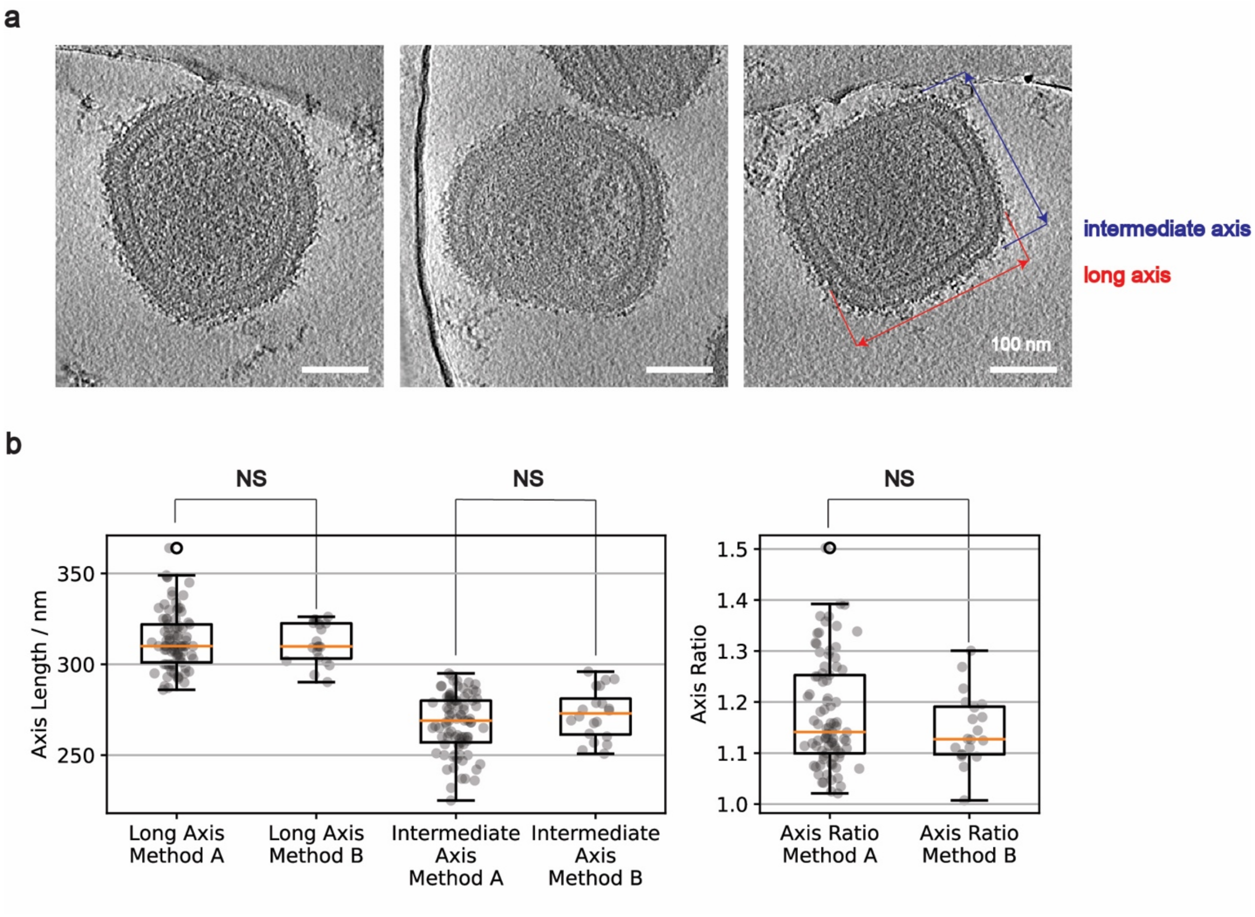

Fig. S2 MPXV purified via two methods is similar in shape and size. **a** Extracellular MVs naturally released were purified without detergent treatment and sonication via Method B in Fig. S1. Thickness of tomogram slices is 27.2 nm. **b** Comparison between particle dimensions and axis ratio of MPXV MVs purified via two methods. Two sample independent t-test was done between measurements of 85 MVs purified via Method A and 20 MVs purified via Method B. NS represents the difference between two samples is not significant (two-sample independent t-test p-value>0.05).

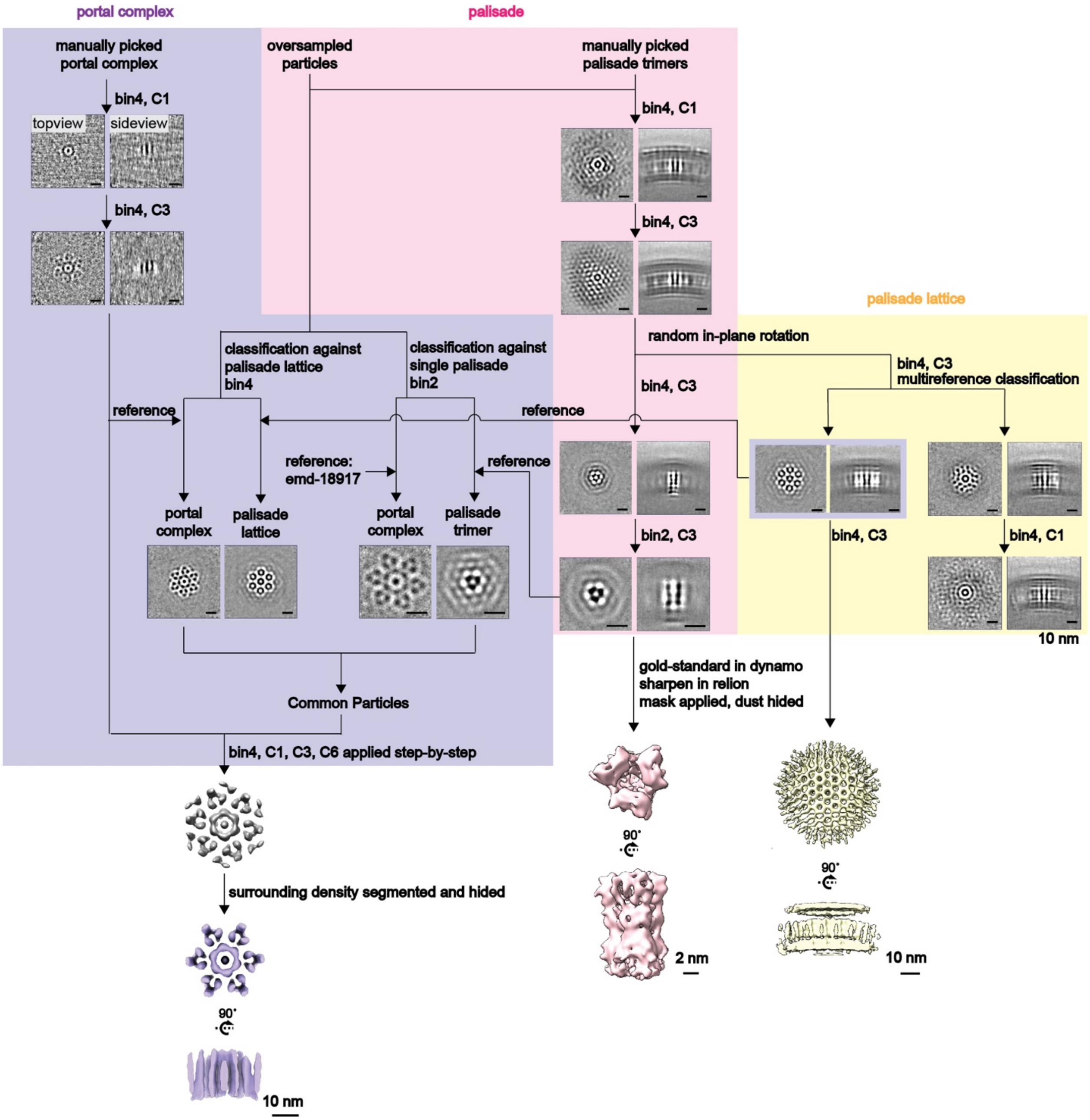

Fig. S3 Summary of sub-tomogram averaging (STA) process. Top view palisade particles were manually picked separately and merged with oversampled particles along core wall for global coarse sub-tomogram aligning with C1 and C3 symmetry (pink box). For single palisade trimer STA, we narrowed the cylindrical mask on the central unit and applied C3 symmetry to obtain a higher resolution map (pink box). The map displayed was further sharpened in relion4 and surrounding density was masked. For STA of palisade lattice, then central subunit and its neighbors

were masked in a larger mask, and multireference STA was applied to classify palisade lattice from randomly-distributed palisades (yellow box). Portal complex structure was initially obtained from manually picked top view particles. To delimitate preferred orientation effect, oversampled particles along core wall was classified with multireference STA and particles classified as portal complex were mixed with manually picked particles for final alignment with symmetry gradually added from C1 to C3 and C6 (purple box). Detailed description of data processing is available in Materials and Method session.

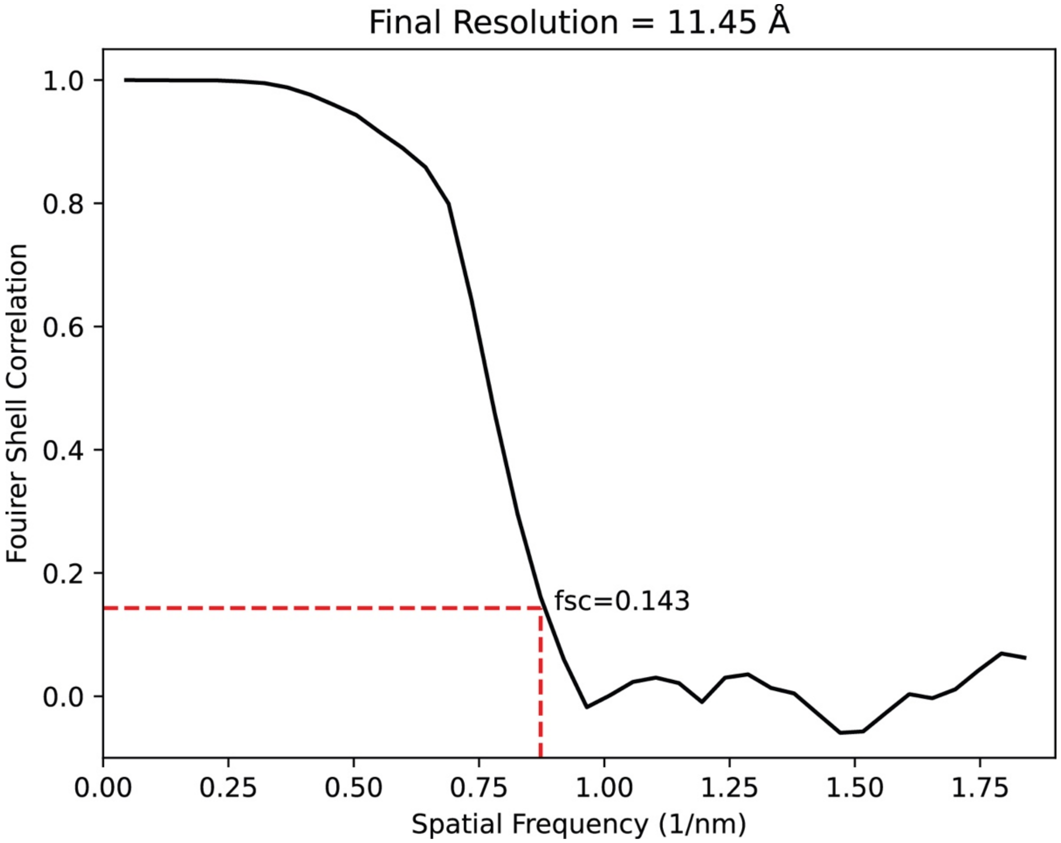

Fig. S4 Fourier shell correlation (FSC) curve of palisade trimer STA structure.

168

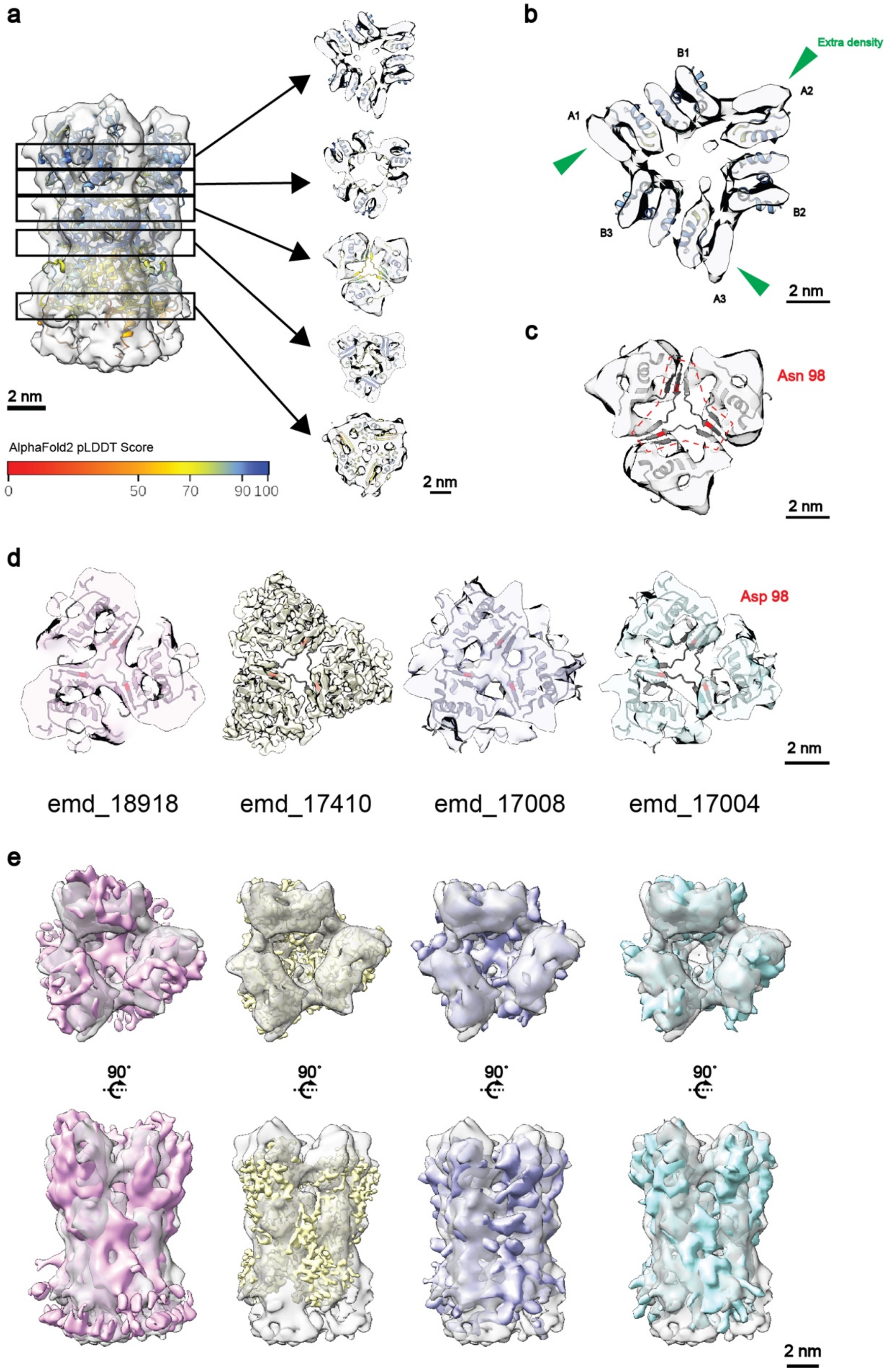
a
2 nm
AlphaFold2 pLDDT Score
0
50
70
90 100
2 nm
b
Extra density
B1
A2
A1
B2
B3
A3
2 nm
c
Asn 98
2 nm
d
Asp 98
2 nm
emd_18918
emd_17410
emd_17008
emd_17004
e
90°
90°
90°
90°
2 nm

Fig. S5 a STA density map and fitted A10 model were sliced at four different transverse planes and zoomed-in for display. The predicted model is colored according to pLDDT score. **b** Magnified top slice in **a**. Extra densities which could not be assigned to the predicted model were indicated with green arrowheads. A1, A2, A3 and B1, B2, B3 represent flank A and B of each three subunits in a palisade trimer. **c** Magnified central slicer in **a**. Red dashed line indicates the predicted connecting density. Amino acid labeld in red is the D98N mutation in MPXV A10. **d** Central slice of previously reported VACV palisade (pink: emd_18918; yellow: emd_17410; purple: emd_17008; blue: emd_17004) [12,19,20] fitted to VACV A10 atomic model (PDB: 8P4K)[19]. Amino acid Asp98 is labeled as red. **e** MPXV palisade STA map is fitted and compared to previously reported VACV palisade (pink: emd_18918; yellow: emd_17410; purple: emd_17008; blue: emd_17004)[12,19,20].

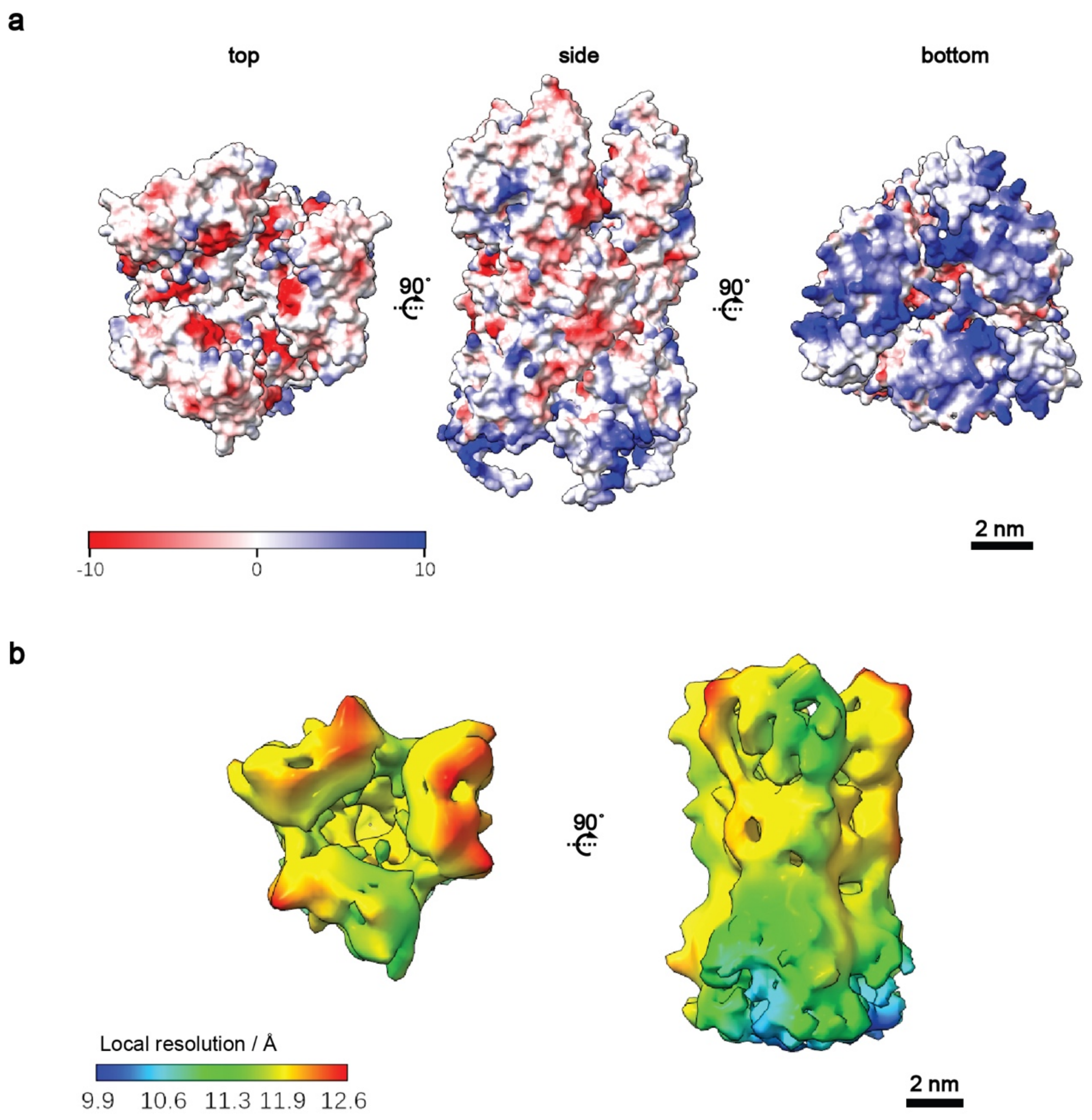

Fig. S6 a Surface charge of predicted palisade trimer. **b** Local resolution of palisade trimer STA density map.

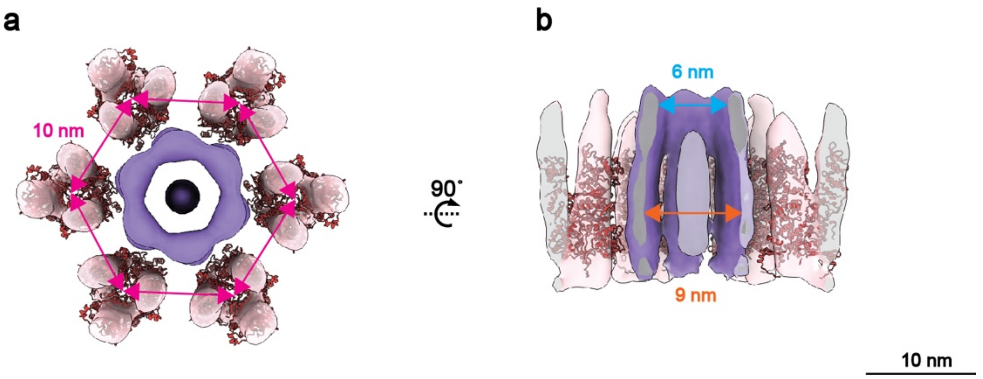

Fig. S7 Portal Complex fitted with surrounding palisade trimers. **a** Top view of the portal complex STA structure. Average distance between palisade trimers in portal complex is 10 nm. **b** Sliced side view of the portal complex STA structure. The narrowest and widest inner diameters of the portal lumen are 6 nm and 9 nm respectively.

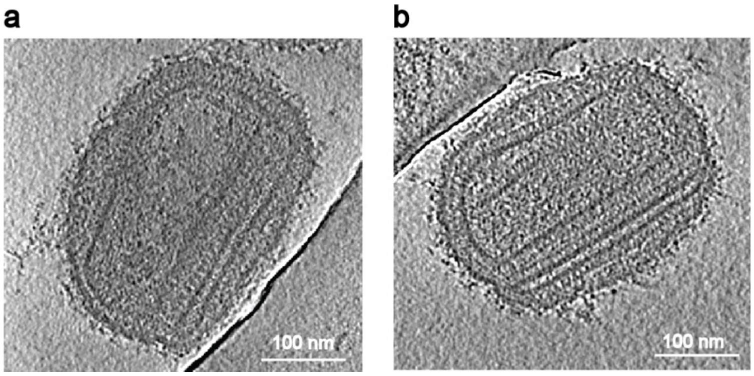

Fig. S8 Irregularly-shaped MV particles from extracellular MPXV that were released naturally. **a** A tomogram slice of MPXV-B.1-China-C-Tan-CQ01 particle isolated without detergent treatment and sonication (Fig S1, method B). Tomogram slice thickness is 27.2 nm. **b** A tomogram slice of MPXV-C.1-China-C-Tan-BJ01 particle (without detergent treatment, sonication or sucrose-gradient ultracentrifugation). Tomogram slice thickness is 19.7 nm.

Supplementary Video S1. An examplary virion and its reconstructed composite structure are shown in movie. Tomogram was lowpassed to 80 Å and virus components were labeled.

Supplementary Video S2. An examplary irregularly-shaped particle with flatten sac and curled lamina is shown in movie. Tomogram was proccessed by isonet and the distorted core walls inside were segmented and labeld.

Supplementary Video S3. An examplary irregularly-shaped particle with multiple small cores and curled lamina is shown in movie. Tomogram was proccessed by isonet and the distorted core walls inside were segmented and labeld.

Table. S1 Cryo-ET data collection and reconstruction statistics.

**Data collection**			
**Microscope**	Titan Krios	**Detector**	Gatan K3
**Magnification**	64,000	**Pixel size (Å)**	0.68 (super-resolution)
**Voltage (kV)**	300	**Energy filter**	Gatan GIF Quantum, 20 eV slit
**Tilt Range**	-51° ~ 51°	**Number of tilts**	35
**Frames per tilt**	10	**Tilt schemes**	Dose-symmetric
**Exposure (e-/Å2)**	137.9	**Defocus range (µm)**	-2.0 ~ -5.5
**Software**	SerialEM		

**Reconstruction and Sub-tomogram Averaging**				
**STA**	Dynamo 1.1.333, Relion 4.0			
**Number of tomograms**	210			
**Number of typical virions for STA**	115			
**Dataset**	Palisade trimer	Honeycomb-like lattice	Less ordered palisade	Portal complex
**Final number of particles**	16,074	10,502	6,073	623
**Symmetry imposed**	C3	C3	C1	C6
**Resolution (Å)**	11.45	N/A	N/A	N/A
**B-factor (Å2)**	-1,766.4	N/A	N/A	N/A
**Final pixel size (Å)**	2.72	5.44	5.44	5.44
**EMDB accession code**	61555	61557	/	61556

References

1 Huang, B. *et al.* Isolation and Characterization of Monkeypox Virus from the First Case of Monkeypox - Chongqing Municipality, China, 2022. *China CDC Wkly* **4**, 1019-1024, doi:10.46234/ccdcw2022.206 (2022).

2 Dou, X. *et al.* Clinical, epidemiological, and virological features of Mpox in Beijing, China - May 31-June 21, 2023. *Emerg Microbes Infect* **12**, 2254407, doi:10.1080/22221751.2023.2254407 (2023).

3 Mastronarde, D. N. Automated electron microscope tomography using robust prediction of specimen movements. *Journal of Structural Biology* **152**, 36-51, doi:10.1016/j.jsb.2005.07.007 (2005).

4 Li, X. M. *et al.* Electron counting and beam-induced motion correction enable near-atomic-resolution single-particle cryo-EM. *Nature Methods* **10**, 584-+, doi:10.1038/nmeth.2472 (2013).

5 Zheng, S. Q. *et al.* MotionCor2: anisotropic correction of beam-induced motion for improved cryo-electron microscopy. *Nat Methods* **14**, 331-332, doi:10.1038/nmeth.4193 (2017).

6 Zhang, K. Gctf: Real-time CTF determination and correction. *J Struct Biol* **193**, 1-12, doi:10.1016/j.jsb.2015.11.003 (2016).

7 Zheng, S. *et al.* AreTomo: An integrated software package for automated marker-free, motion-corrected cryo-electron tomographic alignment and reconstruction. *J Struct Biol X* **6**, 100068, doi:10.1016/j.yjsbx.2022.100068 (2022).

8 Turonova, B., Schur, F. K. M., Wan, W. & Briggs, J. A. G. Efficient 3D-CTF correction for cryo-electron tomography using NovaCTF improves subtomogram averaging resolution to 3.4A. *J Struct Biol* **199**, 187-195, doi:10.1016/j.jsb.2017.07.007 (2017).

9 Castano-Diez, D., Kudryashev, M., Arheit, M. & Stahlberg, H. Dynamo: a flexible, user-friendly development tool for subtomogram averaging of cryo-EM data in high-performance computing environments. *J Struct Biol* **178**, 139-151, doi:10.1016/j.jsb.2011.12.017 (2012).

10 Kremer, J. R., Mastronarde, D. N. & McIntosh, J. R. Computer visualization of three-dimensional image data using IMOD. *J Struct Biol* **116**, 71-76, doi:10.1006/jsbi.1996.0013 (1996).

11 Zivanov, J. *et al.* A Bayesian approach to single-particle electron cryo-tomography in RELION-4.0. *eLife* **11**, e83724, doi:10.7554/eLife.83724 (2022).

12 Hernandez-Gonzalez, M., Calcraft, T., Nans, A., Rosenthal Peter, B. & Way, M. Palisade structure in intact vaccinia virions. *mBio* **0**, e03134-03123, doi:10.1128/mbio.03134-23 (2024).

13 Meng, E. C. *et al.* UCSF ChimeraX: Tools for structure building and analysis. *Protein Sci* **32**, doi:10.1002/pro.4792 (2023).

14 Liu, Y. T. *et al.* Isotropic reconstruction for electron tomography with deep learning. *Nat Commun* **13**, 6482, doi:10.1038/s41467-022-33957-8 (2022).

15 Fedorov, A. *et al.* 3D Slicer as an image computing platform for the Quantitative Imaging Network. *Magnetic Resonance Imaging* **30**, 1323-1341, doi:10.1016/j.mri.2012.05.001 (2012).

16 Berg, S. *et al.* ilastik: interactive machine learning for (bio)image analysis. *Nature Methods* **16**, 1226-1232, doi:10.1038/s41592-019-0582-9 (2019).

17 Stalling, D., Westerhoff, M. & Hege, H.-C. in *The Visualization Handbook.*

18 Gao, M., Nakajima An, D., Parks, J. M. & Skolnick, J. AF2Complex predicts direct physical interactions in multimeric proteins with deep learning. *Nat Commun* **13**, 1744, doi:10.1038/s41467-022-29394-2 (2022).

19 Datler, J. *et al.* Multi-modal cryo-EM reveals trimers of protein A10 to form the palisade layer in poxvirus cores. *Nature Structural & Molecular Biology*, doi:10.1038/s41594-023-01201-6 (2024).

20 Liu, J. *et al.* The palisade layer of the poxvirus core is composed of flexible A10 trimers. *Nature Structural & Molecular Biology*, doi:10.1038/s41594-024-01218-5 (2024).
